# The terpenes camphene and alpha-bisabolol inhibit inflammatory and neuropathic pain via Cav3.2 T-type calcium channels

**DOI:** 10.1186/s13041-021-00876-6

**Published:** 2021-11-14

**Authors:** Vinicius M. Gadotti, Sun Huang, Gerald W. Zamponi

**Affiliations:** Department of Physiology and Pharmacology, Hotchkiss Brain Institute, Alberta Children’s Hospital Research Institute, University of Calgary, AB T2N 4N1 Calgary, Canada

**Keywords:** Pain, Terpenes, Bisabolol, Camphene, T-type, Calcium channels, Cannabis

## Abstract

T-type calcium channels are known molecular targets of certain phytocannabinoids and endocannabinoids. Here we explored the modulation of Cav3.2 T-type calcium channels by terpenes derived from cannabis plants. A screen of eight commercially available terpenes revealed that camphene and alpha-bisabolol mediated partial, but significant inhibition of Cav3.2 channels expressed in tsA-201 cells, as well as native T-type channels in mouse dorsal root ganglion neurons. Both compounds inhibited peak current amplitude with IC50s in the low micromolar range, and mediated an additional small hyperpolarizing shift in half-inactivation voltage. When delivered intrathecally, both terpenes inhibited nocifensive responses in mice that had received an intraplantar injection of formalin, with alpha-bisabolol showing greater efficacy. Both terpenes reduced thermal hyperalgesia in mice injected with Complete Freund’s adjuvant. This effect was independent of sex, and absent in Cav3.2 null mice, indicating that these compounds mediate their analgesic properties by acting on Cav3.2 channels. Both compounds also inhibited mechanical hypersensitivity in a mouse model of neuropathic pain. Hence, camphene and alpha-bisabolol have a wide spectrum of analgesic action by virtue of inhibiting Cav3.2 T-type calcium channels.

**Supplementary Information:**

The online version contains supplementary material available at 10.1186/s13041-021-00876-6.

## Introduction

Cav3.2 T-type calcium channels are important mediators of nociceptive signalling in the primary afferent pain pathway [1, 2] by regulating affent fiber and spinal cord interneuron activity, and synaptic communication in the spinal cord [3–7]. Cav3.2 channel expression is enhanced in a number of painful conditions [8–12], and conversely pharmacological inhibition [13] or molecular biological depletion [14] of these channels has analgesic effects. Hence, it is desriable to identify bioavailable inhibitors of Cav3.2 channels as potential analgesics.

It is well established that cannabinoids derived from cannabis have analgesic properties [15–19]. Although, many are potent agonists of CB1 and/or CB2 cannabinoid receptors, there is evidence that phytocannabinoids such as Delta(9)-Tetrahydrocannabinol (THC) and cannabidiol (CBD) as well as several endocannabinoids also act on other molecular targets, including sodium and TRPV1 channels [20–22]. Cav3.2 calcium channels can be inhibited by certain types of synthetic cannabinoids [23, 24]. They are also potently blocked the endocannabinoids anandamide and N-arachidonoyl glycine [25, 26], suggesting the possibility that the analgesic actions of these endocannabinoids may at least in part be due to inhibition of T-type calcium channels. This is supported by a study by Barbara and colleagues [27] who attributed the analgesic effects of certain endocannabinoids to T-type calcium channel inhibition. Furthermore, THC and CBD block Cav3.1 and Cav3.2 calcium channels [28], altogether revealing a striking action of a range of cannabinoid compounds on T-type calcium channels.

Terpenes are compounds that can give plants their distinctive smell, and a number of different types of terpenes are found in cannabis plants at high concentrations along with cannabinoids [29]. Terpenes alone and in combination with cannabinoids have been shown to exert medicinal effects on patients with anxiety disorders (reviewed in Ref. [30]) and are being explored as possible analgesics in clinical studies (https://clinicaltrials.gov/ct2/show/NCT04451863). We thus wondered if terpenes may also exhibit inhibitory actions on Cav3.2 calcium channels, and if so, whether they may be biologically active in pain models. Here we show that alpha-bisabolol and camphene mediate Cav3.2 channel inhibition and analgesia in mouse models of inflammatory and neuropathic pain.

## Materials and methods

### Cell culture and transient transfection

Human embryonic kidney cells (HEK) tsA-201 cells were grown to 80–90% confluence at 37 °C (5% CO_2_) in Dulbecco’s modified Eagle’s medium (Life Technologies, Grand Island, NY). The medium was supplemented with 10% (vol/vol) fetal bovine serum (Gibco, Thermo Scientific, Pittsburgh, PA), 200 U/ml penicillin, and 0.2 mg/ml streptomycin (Life Technologies, Grand Island, NY). Cells were suspended with 0.25% trypsin/ethylenediaminetetraacetic acid and plated onto glass cover slips in 10 cm culture dishes (Thermo Scientific, Pittsburgh, PA) at 10% confluence 6 h before transfection. The transfections were conducted by a standard calcium phosphate method. For each plate, 5 µg of Cav3.2, Cav3.1 or Cav3.3, and 0.5 µg of green fluorescent protein cDNA were co-transfected. The cells were first cultured at 37 °C and then transferred to 30 °C 16–18 h later following the medium change. The cells were kept at 30 °C for at least two days before recording. Dorsal root ganglion (DRG) neurons were prepared as described by us previously, with the exception that mouse tissue was used in the present study instead of rat [31, 32]. In brief, DRG from 6- to 7-week-old C57 male mice were incubated with 4 mg/ml collagenase (Gibco, Cat#17018-029) and 40 μl/ml papain (Worthington, Cat#LS003126) in culture medium (DMEM (Gibco, Cat#11995) supplemented with 10% heat-inactivated fetal bovine serum (Gibco, Cat#26140-079) and 1% penicillin/Streptomycin (Gibco, Cat#15,140–122)) at 37 °C for 30 min, and then, 1 μg/ml DNase (Sigma, Cat#D5025) at 37 °C for another 10 min, followed by washing and mechanical trituration. Cells were plated on glass coverslips pretreated with Poly-D-Lysine (Sigma, Cat#P7280) and Laminin (Sigma, Cat#L2020) and kept at 37 °C in 5% CO_2_ incubator.

### Whole-cell patch clamp recordings

**Fig. 1:**
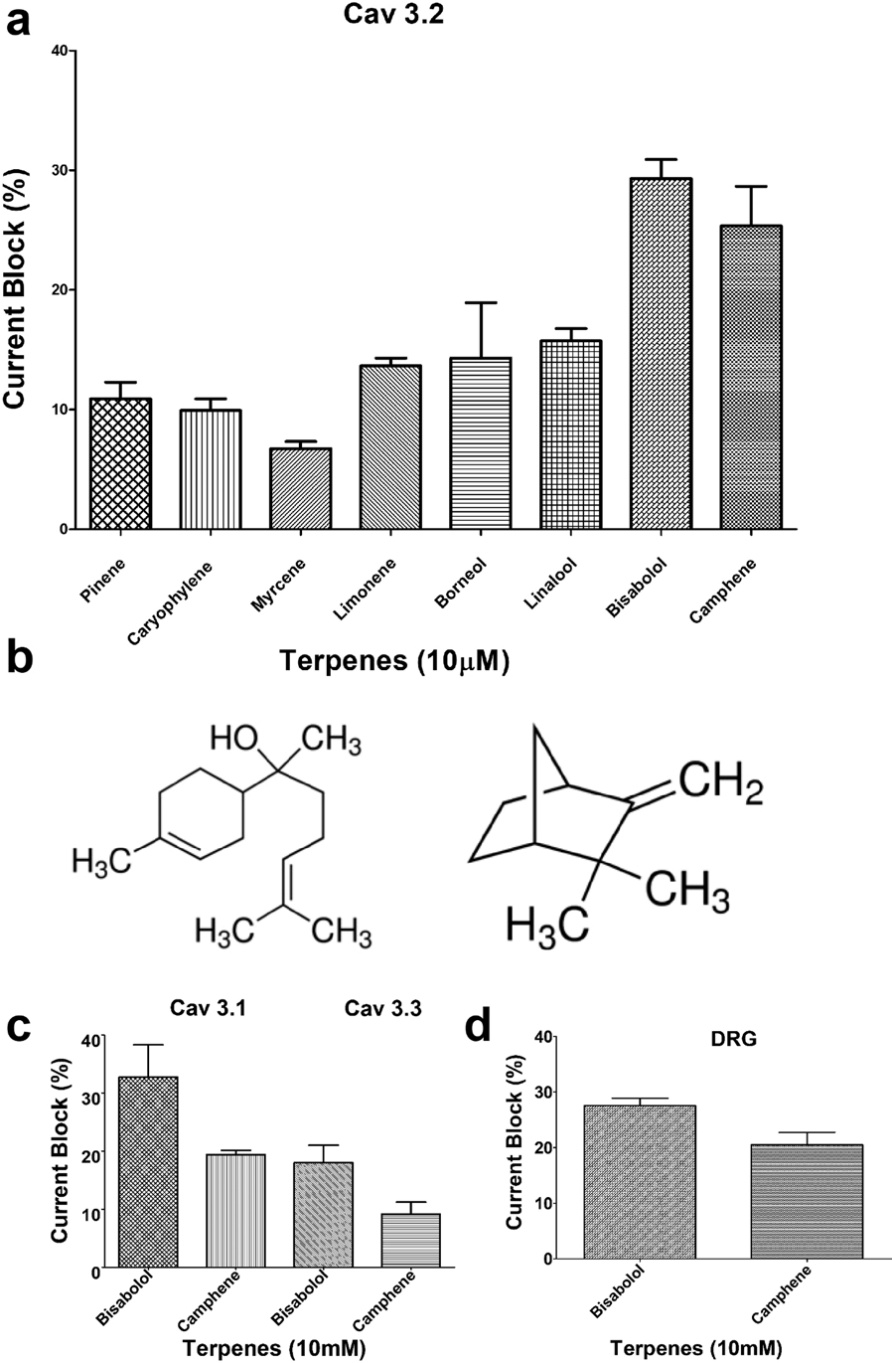
**a** Percentage inhibition of Cav3.2 currents by 10 µM concentrations of terpenes (n = 3). Error bars reflect standard error. **b** Structures of alpha-bisabolol (left) and camphene (right). **c** Percentage inhibition of transiently expressed Cav3.1, Cav3.3 and Cav2.2 calcium channels by 10 µM concentrations of alpha-bisabolol and camphene (n = 3). **d**. Effects of 10 μM alpha-bisabolol and camphene on native T-type currents recorded from cultured mouse DRG neurons (n = 3)

Eight terpenes (Toronto Research Chemicals, see Fig. 1a) were selected for testing on Cav3.2 channels by whole-cell patch clamp recordings. Compounds were prepared as a stock solution of 50 mM. Cells on the glass coverslip were transferred into a 35 mm × 10 mm dish (Corning, Corning, NY) filled with 2 ml of external solution. The external solution contained 40 mM TEACl, 65 mM CsCl, 20 mM BaCl_2_, 1 mM MgCl_2_, 10 mM HEPES and 10 mM glucose, pH was adjusted to 7.4. The glass pipettes (Sutter Instrument Co., Novato, CA, 3–5 MΩ) were filled with internal solution. The internal solution contained 140 mM CsCl, 2.5 mM CaCl_2_, 1 mM MgCl_2_, 5 mM EGTA, 10 mM HEPES, 2 mM Na-ATP and 0.3 mM Na-GTP, pH was adjusted to 7.3. The patch clamp recordings were performed by an EPC 10 amplifier linked to a personal computer equipped with Pulse (V8.65) software (HEKA Elektronik, Bellmore, NY). The recordings were performed when the cells became stable (at least 5 min after break in). The cells were first perfused with external solution to produce stable control currents. Then the cells were perfused with the terpenes dissolved in external solution. Currents were leak corrected with an online P/4 subtraction paradigm. In tsA-201 cells, Cav3.2 currents were elicited by depolarization from a holding potential of − 110 mV to a test potential of -20 mV to 0 mV. Cav3.1 and Cav3.3 currents were elicited by depolarization from a holding potential of − 110 mV to a test potential of − 20 mV. The duration of the test pulse was typically set as 200 ms and the inter-pulse interval was 20 s. For DRG neuron recordings, the holding potential was − 90 mV and the test depolarization was a 100 ms pulse to -30 mV to electrophysiologically isolate T-type currents. Data were recorded at 10 kHz and filtered at 2.9 kHz. Data analysis and graph preparations were completed by GraphPad Prism 5 (GraphPad Software, La Jolla, CA). All electrophysiology data are given as mean values ± standard errors.

### Animals

Experiments were performed after approval of an animal protocol by the Institutional Animal Care and Use Committee and all efforts were made to minimize animal suffering according to the policies and recommendations of the International Association for the Study of Pain. We used male or female C57BL/6 J (wild-type) and male *Cacna1h* (Cav3.2 null, background C57BL/6 J) mice (*mus musculus*, 22–28 g, 7–8 weeks old) that were purchased from the Jackson Laboratory. Animals were housed at a maximum of five per cage (30 × 20 × 15 cm) and with free access to water and food. They were kept in controlled temperature of 23 ± 1 °C on a 12 h light/dark cycles (lights on at 7:00 a.m.) and all experiments were performed between 10 am and 3 pm. Different cohorts of mice were used for each test. The following compounds were used for in vivo studies: Complete Freund`s Adjuvant (CFA) and formalin (Sigma Chemical Company, St. Louis, MO, USA), camphene (Toronto Research Chemicals,Cat No. C165050) and alpha-bisabolol (Toronto Research Chemicals, Cat No. B399808). Mice received 10 μl of camphene, alpha-bisabolol or vehicle intrathecally (i.t.). Appropriate vehicle (DMSO 5% in PBS)-treated groups were assessed simultaneously.

### Formalin test

Mice were acclimatized in the laboratory for at least 60 min before experiments. Animals received 20 μl of a formalin solution (1.25%) made up in PBS injected intraplantarily (i.pl.) in the ventral surface of the right hindpaw. Following i.pl. injections of formalin, the animals were immediately placed individually into observation chambers and the time spent licking or biting the injected paw was recorded and considered as a nociceptive response. We observed animals individually from 0 to 5 min (acute nociceptive phase) and 15–30 min (inflammatory phase) and the time spent licking or biting the injected paw was recorded with a chronometer. Camphene, alpha-bisabolol or vehicle were delivered intrathecally (20 min prior) and their actions on both nociceptive and inflammatory phases were evaluated.

### Persistent inflammatory pain induced by CFA

To induce hyperalgesia produced by peripheral inflammation, 20 μl of Complete Freund's Adjuvant (CFA) was injected subcutaneously in the plantar surface of the right hindpaw (i.pl.) [33]. Sham groups received 20 μl of PBS in the ipsilateral paw. Animals were treated with camphene (3 µg/i.t.), alpha-bisabolol (1 µg/i.t.), or vehicle (10 µl/i.t.) and 3 days following CFA injection they had their thermal withdrawal latency threshold tested.

### Mononeuropathy caused by partial sciatic nerve injury

To induce neuropathic pain in mice, a partial ligation of the sciatic nerve (PSNI) was performed according to Malmberg and Basbaum [34]. Using isoflurane anesthesia and in aseptic conditions the right sciatic nerve was exposed at high-thigh level and a 6–0 silk suture was inserted into the nerve and tightly ligated so that the dorsal 1/3–1/2 of the nerve thickness was trapped in the ligature and the wound was closed with 4–0 silk suture. In all mice the left (contralateral) leg and sciatic nerve were untouched, and in sham operated mice the nerve was left intact. Twenty-one days later the animals received camphene (3 µg/i.t.), alpha-bisabolol (1 µg/i.t.), or vehicle (10 µl/i.t. of PBS). Measurements of mechanical withdrawal thresholds were taken as described below.

### Assessment of thermal and mechanical hypersensitivity

Thermal hyperalgesia of CFA-injected animals was examined by measuring the latency to withdrawal of right hind paws on a focused beam of radiant heat (IR = 30) of a Plantar Test apparatus (UgoBasile, Varese, Italy). Animals were placed individually in a small enclosed testing arena (20 cm × 18.5 cm × 13 cm, length × width × height) on top of a wire mesh floor. Mice were allowed to acclimate for a period of at least 90 min. The device was positioned beneath the animal, so that the radiant heat was directly under the plantar surface of the ipsilateral hind paw. Three trials for each mouse were performed. The apparatus was set at a cut-off time of 30 s to avoid tissue damage. Thermal hyperalgesia was evaluated immediately prior to the treatments (Time 0) and 15, 45, 90 and 180 min after treatment when mice were between 14 and 17 weeks old.

For evaluation of mechanical hyperalgesia of neuropathic mice, we used a digital plantar aesthesiometer (DPA, UgoBasile, Varese, Italy). Mice were placed individually in a small enclosed testing arena (20 cm × 18.5 cm × 13 cm, length × width × height) on top of a grid floor while they acclimated in the experimental room for a period of at least 90 min before the measurements. The aesthesiometer was positioned underneath the animal with the filament directly under the plantar surface of the ipsilateral hind paw. To verify the time-dependence effect of camphene or alpha bisabolol, mechanical withdrawal thresholds were determined at 1 day prior to CFA injection (Baseline), and 3 days after CFA injection at 0, 45, 90 and 180 min after treatment of mice with either camphene or alpha-bisabolol. Each paw was tested three times per session.

### Intrathecal drug treatment

Intrathecal injections were performed in fully conscious mice as previously described [35] and as routinely performed in our lab [36]. Mice were manually restrained, the dorsal fur of each mouse was shaved, the spinal column was arched, and a 30-gauge needle attached to a 25-μl Hamilton microsyringe (Hamilton, Birmingham, UK) was inserted into the subdural space between the L_4_ and L_5_ vertebrae. Accurate positioning of the needle tip was confirmed by a characteristic tail-flick response of animal when the needle if correctly positioned. Intrathecal injections of 10 μl were delivered over a period of 5 s.

### Statistical analysis

For in vivo experiments, data are presented as means ± SEM and evaluated by one-way, two-way or three-way analysis of variance (ANOVA) followed by a Tukey’s test. A value of P < 0.05 was considered to be significant. (^*^P < 0.05; ^**^P < 0.01; ^***^P < 0.001).

## Results

### Cannabis derived terpenes inhibit Cav3.2 channels

We tested the inhibitory actions of eight commercially available terpenes on human Cav3.2 calcium channels transiently expressed in tsA-201 cells using whole cell patch clamp. At a testing concentration of 10 μM, six out of the eight tested terpenes (pinene, caryophylene, myrcene, limonene, borneol and lanalool mediated less than 15 percent inhibition (Fig. 1a). In contrast, alpha-bisabolol (Fig. 1b, left) and camphene (Fig. 1b, right) were twice as effective (29.3% and 25.4% inhibition, repectively). Camphene and alpha-bisabolol were tested on transiently expressed Cav3.1 and Cav3.3 T-type channels (Fig. 1c), revealing that these terpenes block all of these channels, albeit to varying extent with Cav3.3 being the least affected. Finally, we examined the effects of these two terpenes on native T-type currents in cultured mouse DRG neurons, revealing a similar inhibition as that seen with transiently expressed channels (Fig. 1d).

**Fig. 2:**
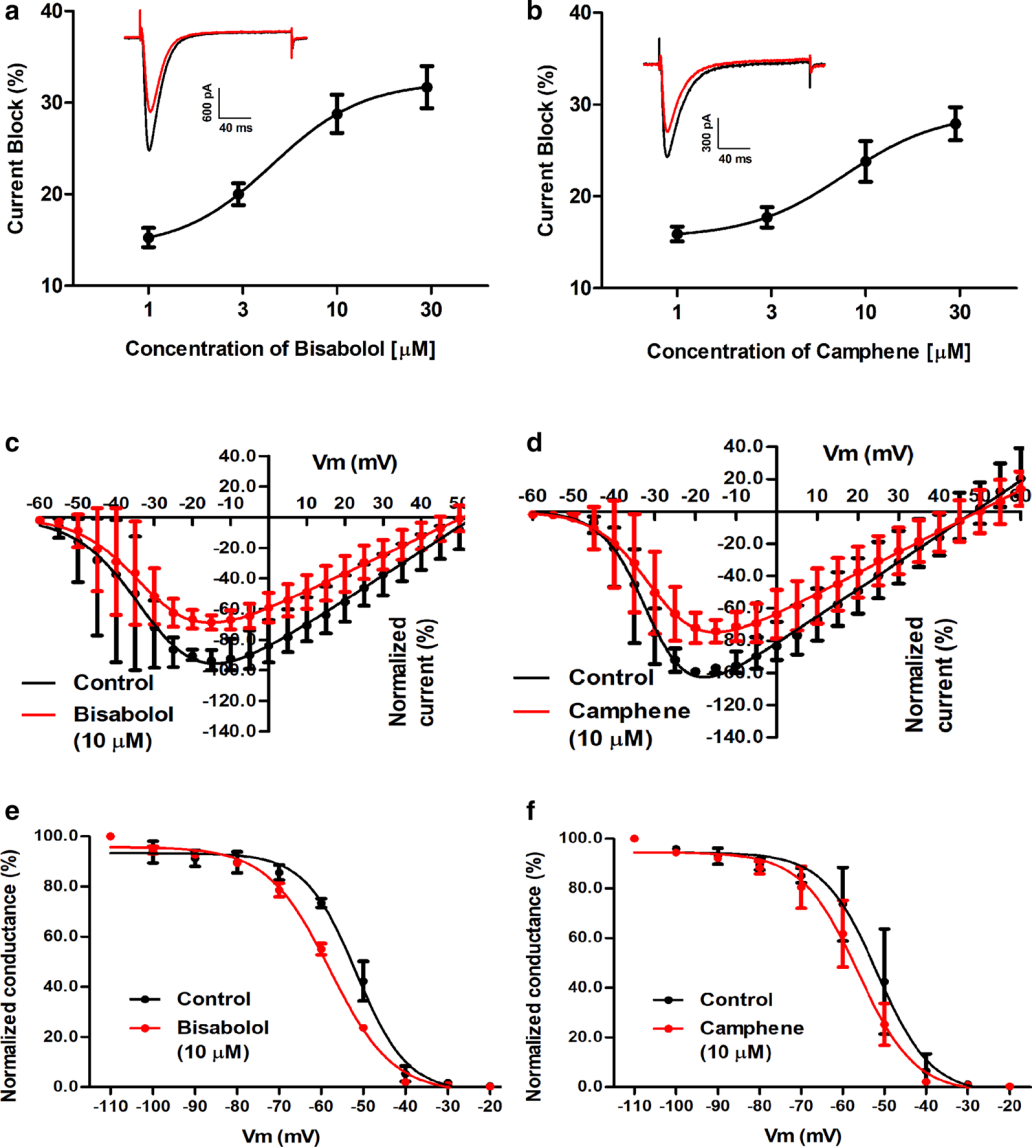
**a** Dose–response relation for alpha-bisabolol inhibition of Cav3.2 channels expressed in tsA-201 cells. The IC_50_ obtained from the fit was 4.52 ± 1.08 µM (n = 3 per dose). The insert shows a representative current before and after the application of 10 µM of alpha-bisabolol. **b** Dose–response relation for camphene inhibition of Cav3.2. The IC_50_ obtained from the fit was 7.73 ± 1.75 µM (n = 3 per dose). The insert shows a representative current before and after the application of 10 µM of camphene. **c** Effect of alpha-bisabolol on the current–voltage relation of Cav3.2. The half activation potentials obtained from the fitted curves were − 30.7 ± 1.9 mV and − 31.2 ± 1.8 mV before and after the application of alpha-bisabolol, respectively (10 µM, n = 3, p = 0.96, Paired *t* test). **d** Effect of camphene on the current–voltage relation of Cav3.2. The half activation potentials obtained from the fitted curves were − 30.9 ± 0.9 mV and − 29.5 ± 1.5 mV before and after the application of camphene, respectively (10 µM, n = 3, p = 0.70, Paired *t* test). **e** Effect of alpha-bisabolol on the steady-state inactivation curve of Cav3.2. The half-inactivation potentials from the fitted curves were − 51.7 ± 0.7 mV and − 57.9 ± 0.4 mV before and after the application of alpha-bisabolol (10 µM, n = 3, p = 0.02, Paired *t* test). **f** Effect of camphene on the steady-state inactivation curve of Cav3.2. The half-inactivation potentials from the fitted curves were − 51.7 ± 1.3 mV and − 56.3 ± 0.9 mV before and after the application of camphene (10 µM, n = 3, p = 0.04, Paired *t* test)

Figure 2a and b depict dose response curves for alpha-bisabolol and camphene inhibition of Cav3.2, indicating that block by these two compounds is partial, with a pleateau of about 30 percent maxiumum inhibition. The IC_50_ values obtained from the dose response curves were 4.5 ± 1.1 μM for alpha-bisabolol, and 7.7 ± 1.8 μM for camphene. Neither compound mediated a significant effect on the voltage-dependence of activation as evident from the current voltage-relations shown in Fig. 2c and d. Bisabalol and to a smaller extent camphene shifted the steady state inactivation curves towards more hyperpolarized potentials (by 6 mV and 4.5 mV, repectively), thus leading to additional inhibitory effects at typical neuronal resting membrane potentials (Fig. 2e, f). For purposes of comparison, we also examined the effects of the phytocannabinoid THC on transiently expressed Cav3.2 channels under the same experimental conditions. As shown in Additional file 1: Fig. S1, THC potently and completely inhibited Cav3.2 channel activity, and like the terpenes, caused a hyperpolarizing shift in the half inactivation potential (Additional file 1: Fig. S1) in line what was reported for this compound previously [28].

Altogether, these data indicate that alpha-bisabalol and camphene are capable of inhibiting recombinant Cav3.2 channels, with bisabalol being more effective.

### Camphene and alpha-bisabolol inhibit inflammatory pain and neuropathic pain

**Fig. 3:**
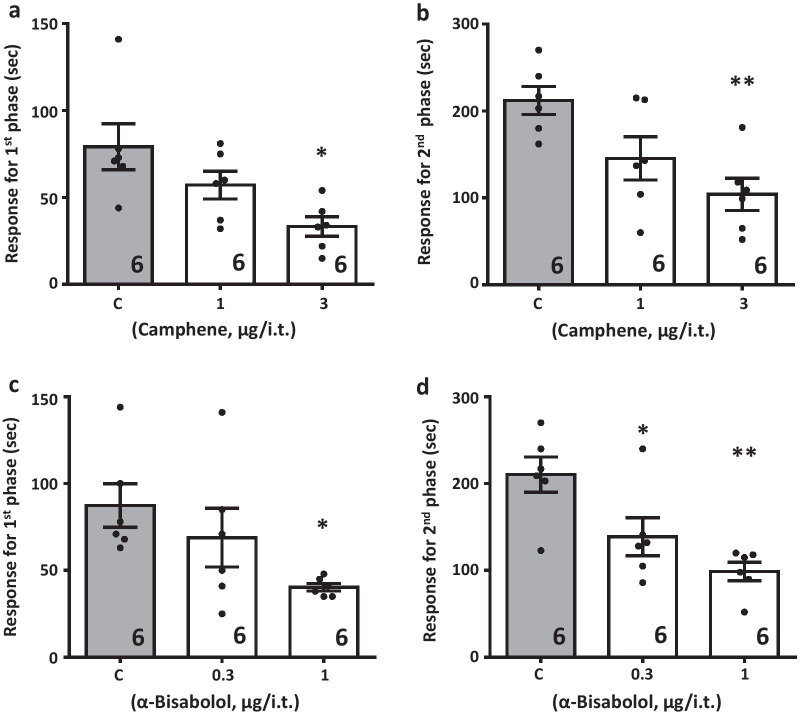
Effect of increasing doses of intrathecal camphene (**a** and **b**) and alpha-bisabolol (**c** and **d**) on the first and second phases of formalin-induced pain. Asterisks denote the significance relative to the control group (*P < 0.05, **P < 0.01, one-way ANOVA followed by Tukey’s test)

**Fig. 4:**
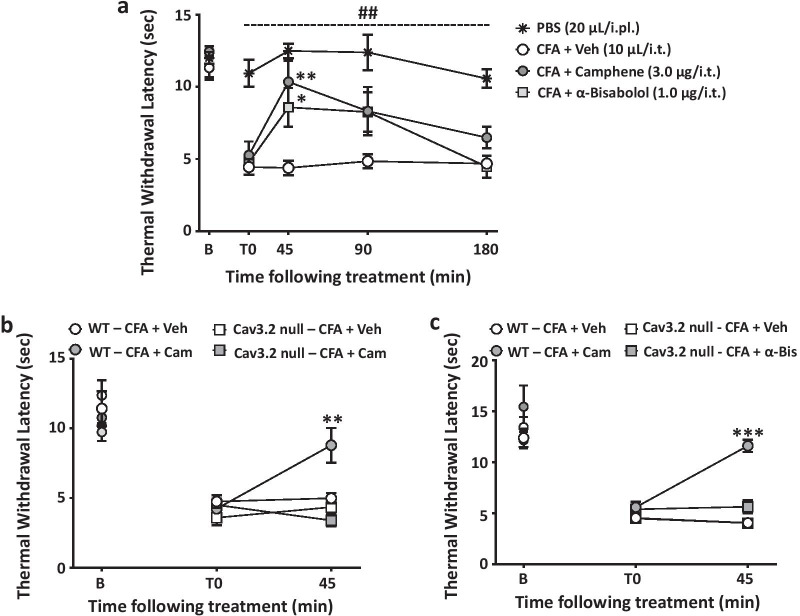
**a** Thermal withdrawal latencies of CFA-injected male mice treated with camphene (3.0 μg/i.t.), alpha-bisabolol (1.0 μg/i.t.) or control vehicle (10 ul/i.t). The dashed line and hashtag indicate the range of data points where PBS treated animals differed from the CFA treated group (## P < 0.01). **b**, **c** Data for thermal hyperalgesia of Cav3.2 null and WT mice when measured 45 min after treatment with **b** camphene (3.0 μg/i.t.) or **c** alpha-bisabolol (1.0 μg/i.t.). Each circle represents the mean ± S.E.M. (n = 4–9) and data are representative of 2 independent sets of experiments. Statistical analyses were performed by two-way, or three-way ANOVA followed by Tukey's test (*P < 0.05, **P < 0.01 relative to vehicle)

**Fig. 5:**
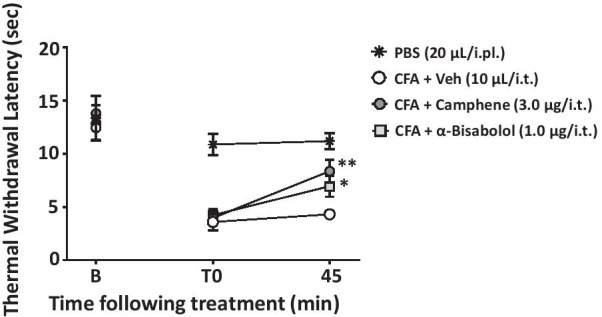
Thermal hyperalgesia of female CFA-treated WT mice when tested 45 min after treatment with either camphene (3.0 μg/i.t.), alpha-bisabolol (1.0 μg/i.t.) or vehicle (10 μl/i.t). Each circle represents the mean ± S.E.M. (n = 6) and data are representative of 3 independent sets of experiments. Statistical analyses were performed by two-way ANOVA followed by Tukey's test (*P < 0.05, **P < 0.01 relative to vehicle)

To ascertain putative in vivo effects of camphene and bisabolol, we first examined their analgesic actions in the formalin test, a model of acute inflammatory pain. Mice received an intraplantar injection of formalin, leading to two distinct phases of nocifensive responses that are characterized by licking and biting of the affected paw. As shown in Fig. 3a–d, camphene and alpha-bisabolol (when delivered intrathecally) reduced the nocifensive response time in both phases in a dose-dependent manner, with bisabolol exerting its effects at a three fold lower dose (in μg) compared to camphene (note that the molecular weight of bisabolol is about 60% greater). Next, we tested the effects of both compounds in a chronic inflammatory pain model based on intraplantar injection of Complete Freund's Adjuvant (CFA). In male mice, CFA injection resulted in a marked reduction in paw withdrawal latency in response to radiant heat (Fig. 4a). When delivered intrathecally, both terpenes increased withdrawal latencies. This effect subsided about 90 min after injection, and was fully reversed at a time point of three hours (Fig. 4a). To determine whether the effects of the two compounds involved an action on Cav3.2 channels, we performed analogous experiments in Cav3.2 null mice. These mice have compensatory mechanisms that allow them to exhibit CFA-induced hypernociception, even though Cav3.2 channels are absent [4]. As shown in Fig. 4b and c, eliminating the putative physiological target eliminated the analgesic effects of camphene and alpha-bisabolol, thus confirming that Cav3.2 channels are required for the in vivo actions of the two terpenes, despite the fact that these compounds inhibit other types of Cav3 channels (see Fig. 1c). There is a large body of evidence supporting sex differences in various aspects of pain signaling [37–40]. To determine if the effects of the two terpenes were subject to sexual dimorphisms, we examined them in a cohort of female mice. As shown in Fig. 5, both camphene and alpha-bisabolol were effective in reducing thermal hypersensitivity in the CFA model, thus indicating that their effects are sex independent.

**Fig. 6:**
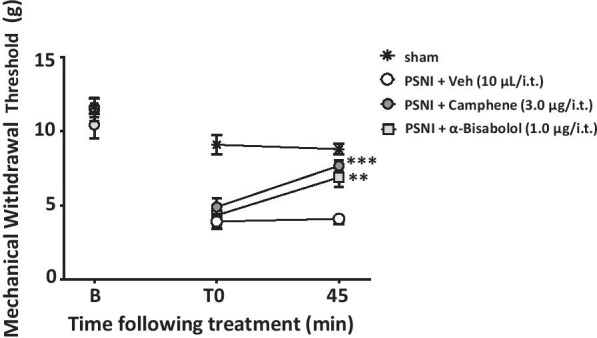
Mechanical withdrawal thresholds of male WT mice subjected to sciatic nerve injury and treated with either camphene (3.0 μg/i.t.), alpha-bisabolol (1.0 μg/i.t.) or vehicle (10 μl/i.t). Each circle represents the mean ± S.E.M. (n = 7) and is representative of 3 independent experiments. (**P < 0.01, ***P < 0.001, two-way ANOVA followed by Tukey’s test relative to vehicle)

To examine whether the two terpenes are effective in neuropathic pain states, we tested them in a mouse model of partial sciatic nerve injury which results in long lasting mechanical hypersensitivity. Injury to the sciatic nerve triggered a robust reduction in mechanical withdrawal threshold that was partially reversed upon intrathecal delivery of camphene or alpha-bisabolol (Fig. 6).

Altogether, these data show that the two terpense examined here have robust effects in mouse models of inflammatory and neuropathic pain.

## Discussion

Natural compounds found in cannabis have been associated with antinociceptive effects [41], most notably for phytocannabinoids such as THC and CBD [15, 18, 19]. Analgesic effects have also been reported for several terpenes. For example, systemic (oral) alpha-bisabolol showed efficacy in a model of visceral inflammation, in the second phase of the formalin test in mice, and in carrageenan induced inflammation [42] (see also [43]). The authors concluded that its effect was mediated by an anti-inflammatory action, rather than an effect on the nervous system. This contrasts with our findings with intrathecal delivery which required T-type channels as a target and most likely an action on neurons. Systemically delivered camphene has also been associated with antinociceptive activity in acetic acid induced writhing behavior, and in the formalin test [44], in agreement with our work. We also note that borneol (one of the compounds that showed poor activity at inhibiting Cav3.2 in our study) mediated analgesic effects in chronic inflammatory and neuropathic pain models in mice [45]. Along these lines, linalool has has been shown to mediate antihyperalgesic activity in a mouse model of fibromyalgia [46]. It is unclear whether these effects involve T-type calcium channels, however, a previous report that showed that linalool reduces neuronal excitability via inhibition of sodium channels [47].

The two compounds studied here have been shown to be present in a variety of cannabis plants and cannabis derived products such as essential oils and bubble gum, including a bisabalool dominant bubble gum version [48]. Alpha-bisabolol and camphene are among the top 20 most abundant tepenes found in 240 different cannabis cultivars of *C. sativa* flower, although typically of much lower abundance than for example myrcene, beta-caryophyllene or d-limonene [49]. Because many other terpenes are found in these cultivars and extracts [48, 49], it is difficult to correlate and biological effects with specific types of terpenes found in these types of products. Many terpenes are able to cross the blood brain barrier [50, 51], and hence it is unclear whether the systemic effects described in the above studies are mediated by CNS or peripheral actions. In our study, camphene and alpha-bisabolol were delivered exclusively via the intrathecal route, making it highly unlikely that their actions occurred at the level of the brain, as opposed to inhibiting T-type channel activity either in spinal cord neurons, or at nerve endings of primary afferent fibers. Our data with Cav3.2 null mice clearly indicate that the analgesic properties of spinally delivered terpenes required an action on Cav3.2 channels which are known to be expressed in specific afferents and in the spinal cord [5, 6].

In our hands, in contrast with THC, the effects of both terpenes were partial, with a plateau of just over 30% inhibition of Cav3.2 channels at a holding potential of − 100 mV. Additional inhibition could be observed due to a small, but significant shift in half-inacivation potential, leading to reduced availablity of channel opening at more depolarized resting membrane potentials. Is is unclear why the inhibitory effects are partial. Possiblities include partial occlusion of the pore of the channel, or an allosteric effect that may result in a reduction in the maximum open probability of the channel without affcting permeation. Irrespective of how the compounds inhibit the function of the channel at the molecular level, the combined effects of resting channel block and the effect on the voltage-dependence of inactivation culminate in aproximately 50% inhibition of channel activity at saturating doses. Assuming that native channels behave similarly to those expressed in tsA-201 cells, such a reduction in overall Cav3.2 activity should be sufficient to account for the observed analgesic effects.

## Conclusions

In summary, we report a Cav3.2 channel dependent analgesic effect of two terpenes that are present in cannabis plants. To what extent this contributes to cannabis mediated analgesia needs to be explored further.

## Supplementary Information

**Additional file 1: Fig. S1.** Effect of THC on Cav3.2 calcium channels. Dose response curve for THC block of Cav3.2 channels, and effect of THC on activation and inactivation of the channels.

## Data Availability

All data generated or analysed during this study are included in this published article.

## Abbreviations

THC: Delta(9)-tetrahydrocannabinol
CBD: Cannabidiol
CFA: Complete Freund’s adjuvant
PBS: Phosphate buffered saline
DRG: Dorsal root ganglion
