# Supplementary material for: The terpenes camphene and alpha-bisabolol inhibit inflammatory and neuropathic pain via Cav3.2 T-type calcium channels
Source: Mol Brain. 2021 Nov 14;14:166. doi: 10.1186/s13041-021-00876-6 (PMC8591808; doi:10.1186/s13041-021-00876-6)
Supplement: Supplementary file 1 — Additional file 1: Fig. S1. Effect of THC on Cav3.2 calcium channels. Dose response curve for THC block of Cav3.2 channels, and effect of THC on activation and inactivation of the channels. [file 13041_2021_876_MOESM1_ESM.pdf]

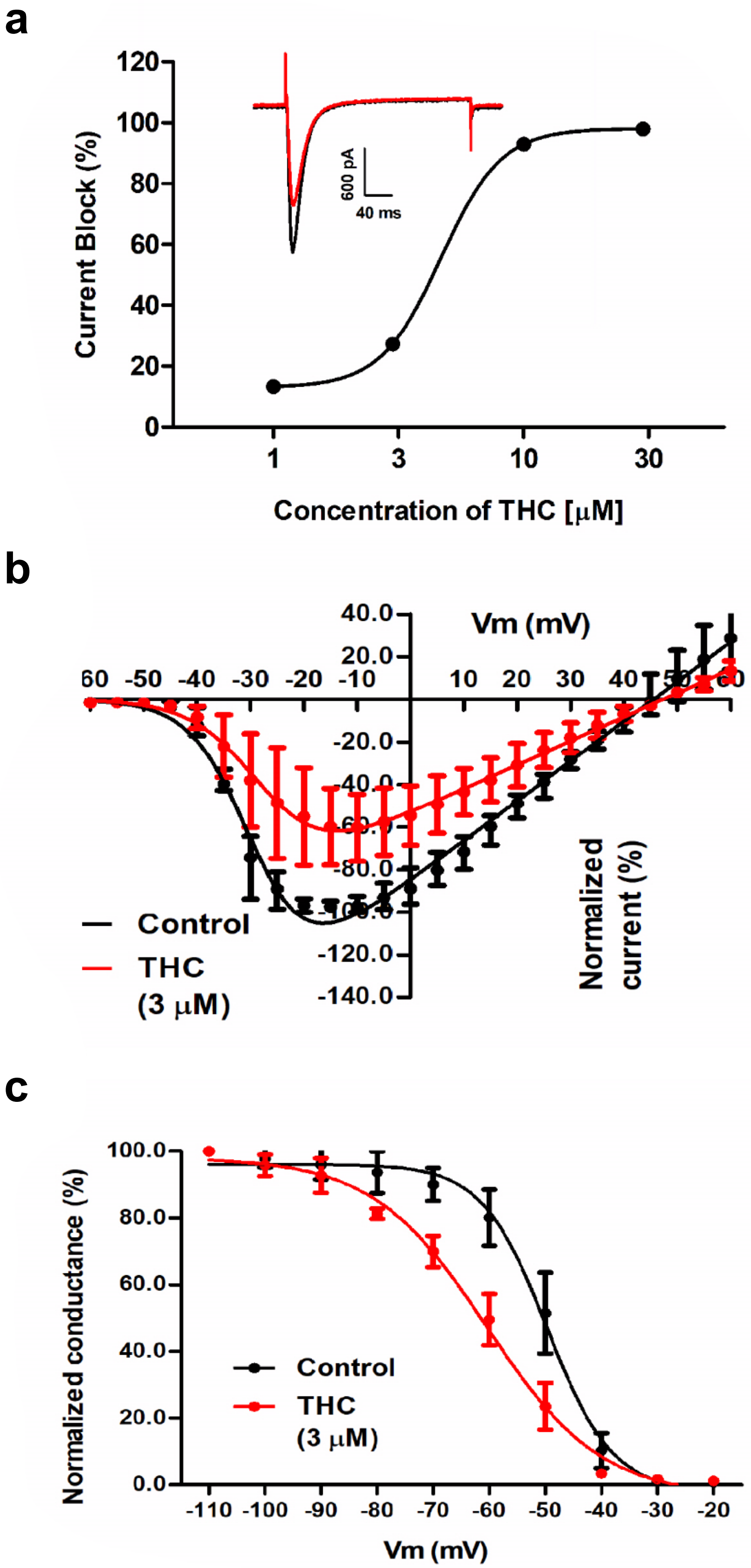

a
Current Block (%)
Concentration of THC [μM]
600 pA
40 ms
b
Vm (mV)
Normalized current (%)
Control
THC (3 μM)
c
Normalized conductance (%)
Vm (mV)
Control
THC (3 μM)

Figure S1: Supplemental figure examining the effects of THC on Cav3.2 channels.
a) Dose-response relation for THC inhibition of $Ca_V3.2$. The IC_{50} obtained from the fit was 4.67 ± 0.19 μM (n = 4 per dose). The insert shows a representative current before and after the application of 3 μM of THC. **b)** Effect of THC on the current–voltage relation of $Ca_V3.2$. The half activation potentials obtained from the fitted curves were -29.1 ± 0.5 mV and -27.3 ± 1.3 mV before and after the application of THC, respectively (3 μM, n = 4, p = 0.25, Paired *t* test). **c)** Effect of THC on the steady-state inactivation curve of $Ca_V3.2$. The half-inactivation potentials from the fitted curves were -49.7 ± 0.7 mV and -60.4 ± 0.9 mV before and after the application of THC (3 μM, n = 4, p = 0.004, Paired *t* test).
